# Low temperature methanation of CO_2_ over an amorphous cobalt-based catalyst†

**DOI:** 10.1039/d0sc06414a

**Published:** 2021-01-15

**Authors:** Jinghui Tu, Haihong Wu, Qingli Qian, Shitao Han, Mengen Chu, Shuaiqiang Jia, Ruting Feng, Jianxin Zhai, Mingyuan He, Buxing Han

**Affiliations:** Shanghai Key Laboratory of Green Chemistry and Chemical Processes, School of Chemistry and Molecular Engineering, East China Normal University Shanghai 200062 P. R. China hhwu@chem.ecnu.edu.cn hanbx@iccas.ac.cn; Beijing National Laboratory for Molecular Sciences, CAS Key Laboratory of Colloid and Interface and Thermodynamics, CAS Research/Education Center for Excellence in Molecular Sciences, Institute of Chemistry, Chinese Academy of Sciences China qianql@iccas.ac.cn

## Abstract

CO_2_ methanation is an important reaction in CO_2_ valorization. Because of the high kinetic barriers, the reaction usually needs to proceed at higher temperature (>300 °C). High-efficiency CO_2_ methanation at low temperature (<200 °C) is an interesting topic, and only several noble metal catalysts were reported to achieve this goal. Currently, design of cheap metal catalysts that can effectively accelerate this reaction at low temperature is still a challenge. In this work, we found that the amorphous Co–Zr_0.1_–B–O catalyst could catalyze the reaction at above 140 °C. The activity of the catalyst at 180 °C reached 10.7 mmol_CO_2__ g_cat_^−1^ h^−1^, which is comparable to or even higher than that of some noble metal catalysts under similar conditions. The Zr promoter in this work had the highest promoting factor to date among the catalysts for CO_2_ methanation. As far as we know, this is the first report of an amorphous transition metal catalyst that could effectively accelerate CO_2_ methanation. The outstanding performance of the catalyst could be ascribed to two aspects. The amorphous nature of the catalyst offered abundant surface defects and intrinsic active sites. On the other hand, the Zr promoter could enlarge the surface area of the catalyst, enrich the Co atoms on the catalyst surface, and tune the valence state of the atoms at the catalyst surface. The reaction mechanism was proposed based on the control experiments.

## Abstract 2

It is discovered that an amorphous transition metal catalyst Co–Zr0.1–B–O could effectively accelerate CO2 methanation, at a rate that is comparable to or even higher than that of some noble metal catalysts under similar conditions.

## Graphical abstract

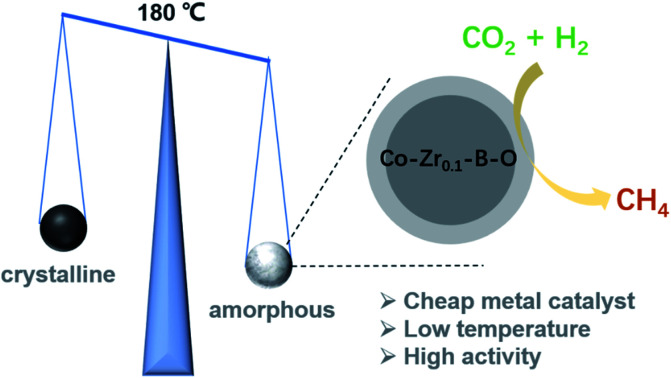

## Introduction

CO_2_ is a well known greenhouse gas, while it is also a cheap, nontoxic, and renewable carbon resource. Chemical transformation of CO_2_ into fuels or useful chemicals has attracted great attention all over the world.^1–10^ Methane is one of the major energy sources in human life that can be easily fed into the existing infrastructures. In addition, methane is also a basic feedstock to produce other value added chemicals.^11–13^ Hydrogenation of CO_2_ into methane, *i.e.*, CO_2_ methanation, is among the most important topics of CO_2_ valorization.^14,15^ CO_2_ methanation is a reversible and strong exothermic reaction, and is thermodynamically favorable. However, it is difficult to achieve because of the high kinetic barriers of the eight-electron reduction process. Many transition metals such as Ni, Fe, Co, Ru, Rh, and Pd have been investigated as catalysts to accelerate this reaction.^16–19^ To obtain satisfactory catalytic results, the reaction usually needed to proceed at higher temperature (>300 °C), where the undesired endothermic reverse water gas shift (RWGS) reaction tended to occur. For example, nickel based catalysts with various supports were the extensively studied catalysts for CO_2_ methanation, which usually operated at 300–350 °C.^20–23^ Cobalt or iron based catalysts have also been widely investigated for CO_2_ methanation, and satisfactory performances were generally obtained at 400 °C or higher.^24–28^

Low temperature catalysis is still one of the major challenges in methanation of CO_2_. Design of catalysts that can work effectively at lower temperature has received considerable attention.^29^ Although many efforts have been made, the progress was restricted to several noble metal catalysts, especially at a temperature below 200 °C.^30–36^ Obviously, low temperature methanation of CO_2_ over cheap metal catalysts is highly desirable. Herein we show that the amorphous Zr-doped Co–B–O catalyst can effectively accelerate the CO_2_ methanation at above 140 °C. Excellent activity was obtained at 180 °C, which is comparable to or even higher than those of some noble metal catalysts (Table S1†). Moreover, the addition of Zr promoter results in the highest promoting factor to date of the catalysts for CO_2_ methanation. No CO was observed under all conditions and the reaction was not *via* the RWGS pathway. To our knowledge, this is the first report of an amorphous transition metal catalyst that can effectively accelerate CO_2_ methanation.

## Results and discussion

### The catalyst

**Fig. 1:**
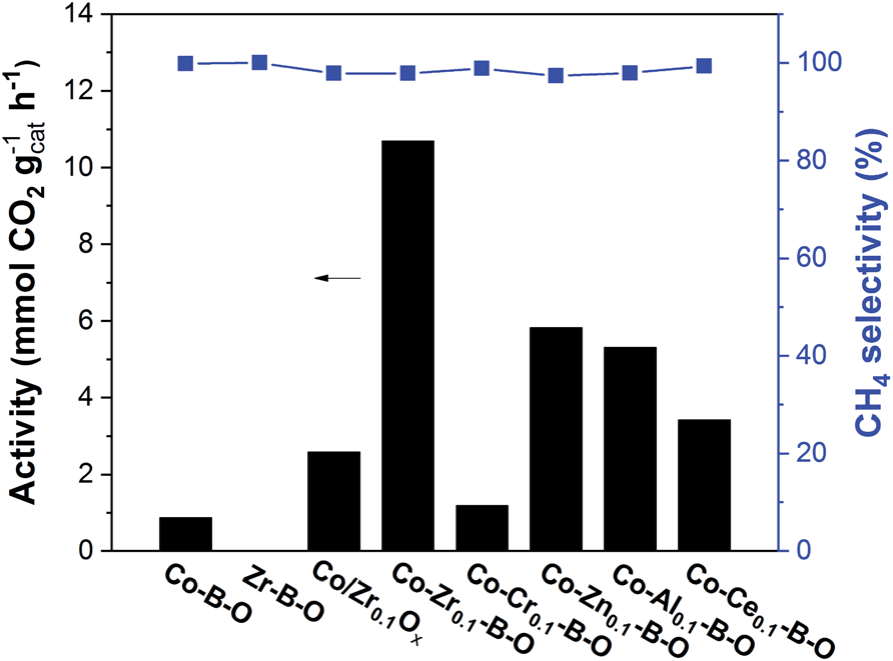
Catalytic results of different catalysts. Conditions: catalyst 40 mg, cyclohexane 2 mL, 4 MPa CO_2_, 4 MPa H_2_, 180 °C, 12 h.

The Co–Zr_0.1_–B–O catalyst was prepared by a liquid phase reduction method using NaBH_4_ as the reductant in the presence of ammonia. The reaction results over different catalysts at 180 °C are shown in Fig. 1. The selectivities of all the catalysts were high (>97%), while the catalytic activities of different catalysts varied significantly. The activity of the Co–Zr_0.1_–B–O catalyst was as high as 10.7 mmol_CO2_ g_cat_^−1^ h^−1^. The yield of methane was 78.1% and the methane selectivity was 97.8%, with minor C_2+_ hydrocarbons as byproducts. In contrast, the activity of the Co–B–O catalyst was merely 0.87 mmol_CO2_ g_cat_^−1^ h^−1^, and the activity of the Zr–B–O catalyst was negligible. These indicated that remarkable synergy existed between Co and Zr in the Co–Zr_0.1_–B–O catalyst. The promoting factor, which indicates the ratio of the catalytic activity of the promoted catalyst to that of the non-promoted catalyst, was usually adopted to compare the impact of the promoter on the catalytic performance. In the previous reports of CO_2_ methanation, the average promoting factor was about 3.0.^29^ The addition of noble Pt to the Co nanocatalyst could enhance the catalytic activity by a factor of 6.^37^

In our work, the promoting factor was as high as 12.3, which is remarkably higher than those of the reported catalysts. The Co/Zr_0.1_O_*x*_ catalyst was prepared by the commonly used method, *i.e.*, coprecipitation, calcination followed by reduction with H_2_ at high temperature (400 °C). But its catalytic activity was much lower than that of the Co–Zr_0.1_–B–O catalyst. Using the liquid phase reduction method, we also prepared the catalysts with other promoters (Cr, Zn, Al, and Ce). Most of them (Co–Zn_0.1_–B–O, Co–Al_0.1_–B–O, and Co–Ce_0.1_–B–O) were also more effective than the Co/Zr_0.1_O_*x*_ catalyst, but they were markedly less efficient than the Co–Zr_0.1_–B–O catalyst. In short, Co–Zr_0.1_–B–O was an outstanding catalyst for low temperature CO_2_ methanation.

### The catalyst characterization

**Fig. 2:**
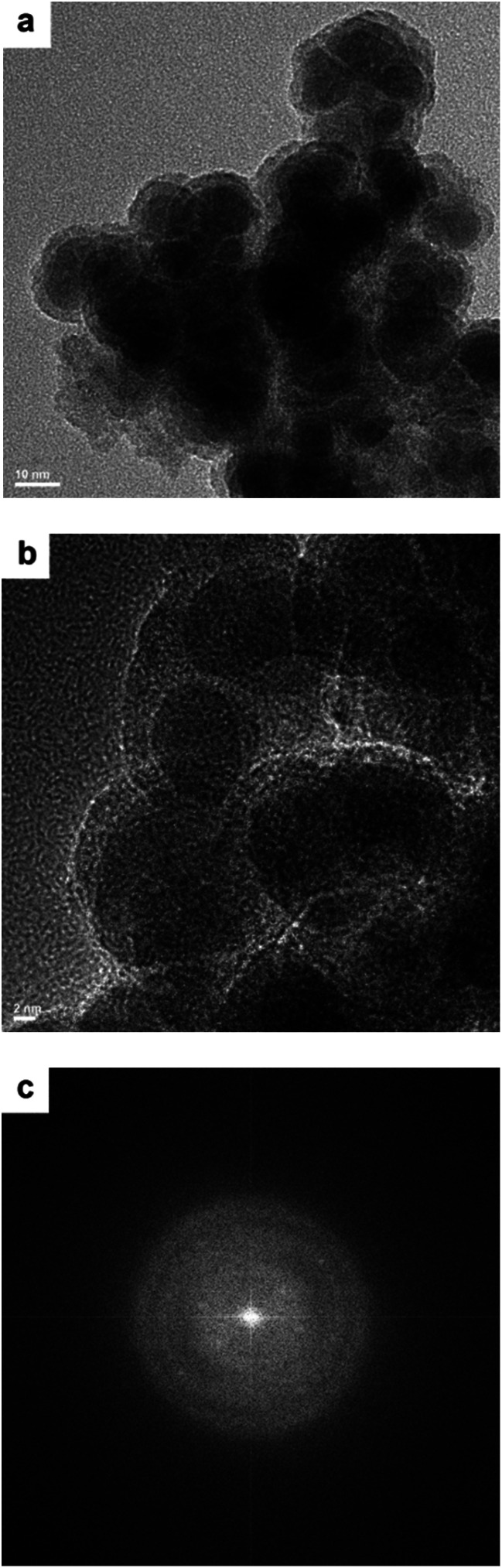
TEM images (a and b) and the SAED pattern (c) of the Co–Zr_0.1_–B–O catalyst.

**Fig. 3:**
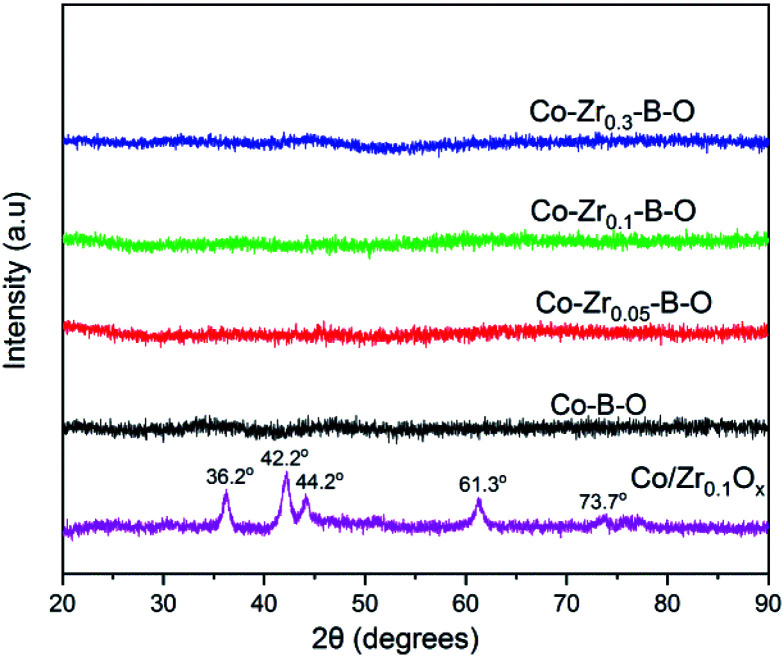
XRD patterns of different catalysts.

The TEM images of the Co–Zr_0.1_–B–O catalyst are given in Fig. 2a and b. The catalyst was mainly composed of 5–15 nm spherical like particles, the outer layers of which seemed different from the cores. No crystal lattice was observed in the TEM images, indicating that the catalyst had an amorphous structure. No diffraction ring was observed in the selected area electron diffraction (SAED) pattern of the catalyst either, which agrees with the TEM images (Fig. 2c). The XRD pattern showed that the Co–Zr_0.1_–B–O catalyst had no discernible diffraction peak, which further confirmed that it was amorphous (Fig. 3). Actually, all the catalysts prepared by the liquid phase reduction method were amorphous. In contrast, the XRD curve of the Co/Zr_0.1_O_*x*_ catalyst displayed remarkable diffraction peaks. The peaks at 36.2°, 42.2°, 61.3° and 73.7° were attributed to the (111), (200), (220) and (311) planes of CoO, and the peak at 44.2° was ascribed to Co (111). The results of N_2_ adsorption test of the catalysts are shown in Fig. S1.† The adsorption isotherm of the Co–Zr_0.1_–B–O catalyst can be classified as a type III curve, suggesting that multilayer adsorption occurred on the lyophobic surface. The Brunauer–Emmett–Teller (BET) surface area was 92.4 m^2^ g^−1^, indicating that it is not a porous material. The results of the EDS elemental mapping revealed that the Co, Zr, B, and O atoms were well dispersed in the catalyst (Fig. S2†). The X-ray photoelectron spectroscopy (XPS) characterization suggests that different Co species, *i.e.*, Co^0^, Co^2+^ and Co–OH, existed on the catalyst surface (Fig. S3†). The FTIR spectra demonstrated the presence of the OH group on the catalyst, which coincides with the XPS result (Fig. S4†).

### Impact of reaction conditions

**Fig. 4:**
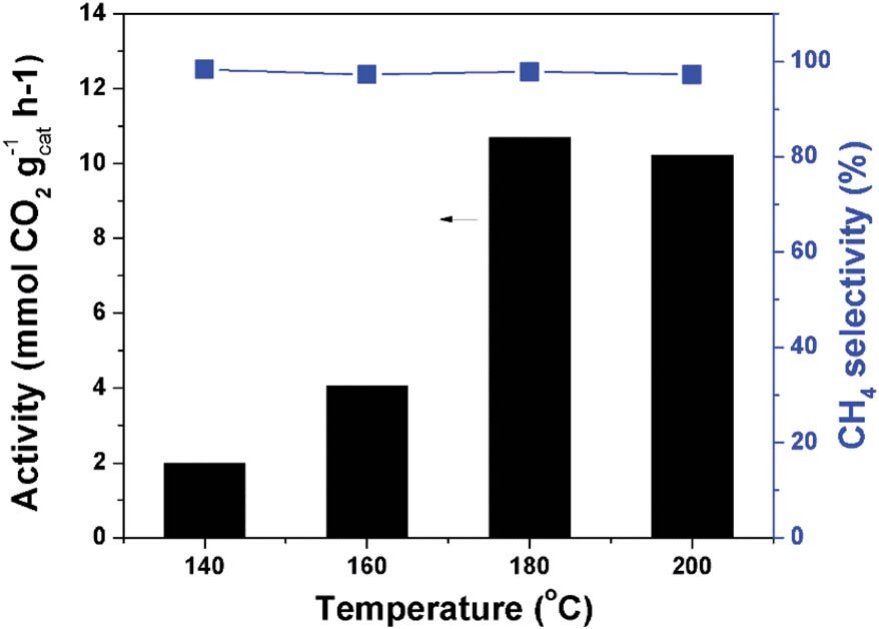
Catalytic results at different temperatures. Conditions: catalyst 40 mg, cyclohexane 2 mL, 4 MPa CO_2_, 4 MPa H_2_, 12 h.

Fig. 4 depicts the catalytic results at different temperatures. The reaction could occur at 140 °C. The catalytic activity increased quickly with increasing temperature until 180 °C. When the temperature was further increased the reaction rate can not be effectively improved. This could be explained by the partial transformation of the amorphous catalyst structure. The XRD analysis demonstrated that obvious crystals of cobalt were formed when the catalyst was treated at higher temperature (Fig. S5†). The peaks at 44.2° and 47.3° are ascribed to Co (111) and Co (101), respectively. The methane selectivity was very high and remained nearly constant at different temperatures. Without the precharged CO_2_ and/or H_2_ no product was detected after the reaction, demonstrating that both CO_2_ and H_2_ took part in the reaction. The reaction started by adsorption of CO_2_ and H_2_ at the catalyst surface. The ratio of CO_2_ and H_2_ pressures remarkably affected the reaction results, and the suitable ratio was 1/1 (Fig. S6†). We fixed this ratio and conducted the reaction at different total pressures. As expected, the reaction rate increased with increasing pressure, and the increase became slow when pressure was high enough (Fig. S7†). The adsorption of the reactants CO_2_ and H_2_ on the catalyst surface was remarkably enhanced by increasing their pressure, which agreed with the results of the N_2_ adsorption test. The catalytic performance could also be tuned by the solvent effect. We conducted the reaction in different solvents and cyclohexane was proved to be an appropriate solvent (Fig. S8†). At the optimized temperature and pressure, we carried out the time course study. It was shown that the reaction rate was very quick at the beginning and it gradually slowed down with the consumption of H_2_ (Fig. S9†).

### Effect of the Zr promoter

**Fig. 5:**
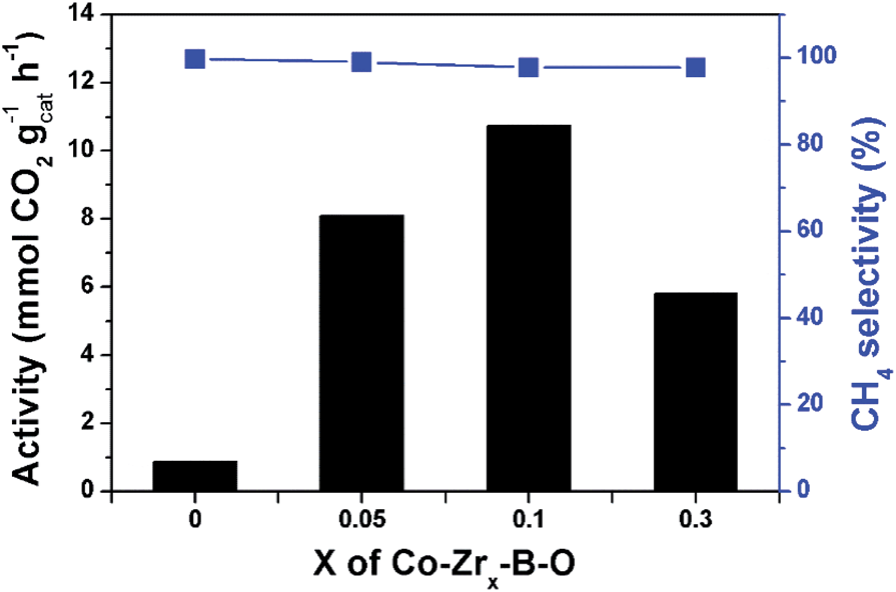
Effect of the Zr content in the catalyst on the reaction. Conditions: catalyst 40 mg, cyclohexane 2 mL, 4 MPa CO_2_, 4 MPa H_2_, 180 °C, 12 h. *X* represents the molar ratio of Zr/Co, *i.e.*, *n*_Zr_/*n*_Co_.

As revealed in Fig. 1, the Zr–B–O catalyst could not promote the reaction, and addition of Zr promoter to the Co–B–O catalyst could significantly accelerate the reaction activity. The Zr content in the catalyst obviously affected the catalytic activity, as depicted in Fig. 5. With increasing molar ratio of the Zr promoter (*n*_Zr_/*n*_Co_: 0.05, 0.1, and 0.3), the reaction rate increased markedly and reached a maximum at 0.1. When the *n*_Zr_/*n*_Co_ was further increased the reaction activity decreased. This suggests that *n*_Zr_/*n*_Co_ = 0.1 was an appropriate ratio. The role of the Zr promoter in modulating the catalyst structure may be ascribed mainly to three aspects, *i.e.*, changing the surface area of the catalyst, enriching the Co atoms on the catalyst surface, and varying the valence of the surface atoms. The BET surface areas and surface compositions of the Co–B–O catalyst and the Zr doped Co–B–O catalysts are given in Table S2.† The results indicated that the surface area of the Co–B–O catalyst (18.8 m^2^ g^−1^) could be significantly enhanced by the Zr promoter. The surface area of the Co–Zr_0.1_–B–O catalyst was 92.4 m^2^ g^−1^, which was the highest surface area in the Zr doped catalysts. It is equally important that the Zr promoter could greatly enrich the active Co atoms on the catalyst surface. The Co content in total surface atoms of Co–B–O was 7.7%, while it reached above 30% in Co–Zr_0.05_–B–O and Co–Zr_0.1_–B–O catalysts. The synthesis of the Co–B–O catalyst is an exothermic process, which involves high surface energy and tends to cause agglomeration.^38^ The addition of the Zr promoter could affect the fabrication of the catalyst. Higher surface area and Co enrichment on the surface may cooperatively increase the active sites for the reaction. However, when excess Zr promoter was added, both the surface area and surface Co content of the catalyst decreased. This may explain partially why the performance of the Co–Zr_0.3_–B–O catalyst was not as good as that of the Co–Zr_0.1_–B–O catalyst. Besides the impact on the structure, the Zr promoter could also alter the valence of the surface atoms (Fig. S3†). The Zr atoms mostly existed as Zr^4+^ in the catalyst, which did not change during the fabrication of the catalyst. With increasing Zr promoter, the Co atoms shifted to higher oxidation states. It is noteworthy that the lattice O atoms doped in the catalyst were greatly increased by adding the Zr promoter (Table S3†). It was reported that the oxygen atoms doped in cobalt metal may create surface defects, which acted as overactive sites and enhanced the rate of the catalytic reactions, including CO_2_ methanation.^39,40^ Besides the doped O atoms, the amorphous structure may further increase the surface defects.^41^ In short, the Zr in the catalyst acted as a structural promoter and an electronic promoter simultaneously, and the Co–Zr_0.1_–B–O was the optimal catalyst.

### The reusability of the catalyst

The recycling test of the Co–Zr_0.1_–B–O catalyst was conducted to appraise its reusability. After the reaction, the residual gases were analyzed and released, and the catalyst was used directly for the next run. The results of the recycling test indicated that the catalytic performance had no obvious decrease after five cycles (Fig. S10†). The elements of the catalyst were still well dispersed after the reaction (Fig. S11†).

### Mechanistic discussion

**Fig. 6:**
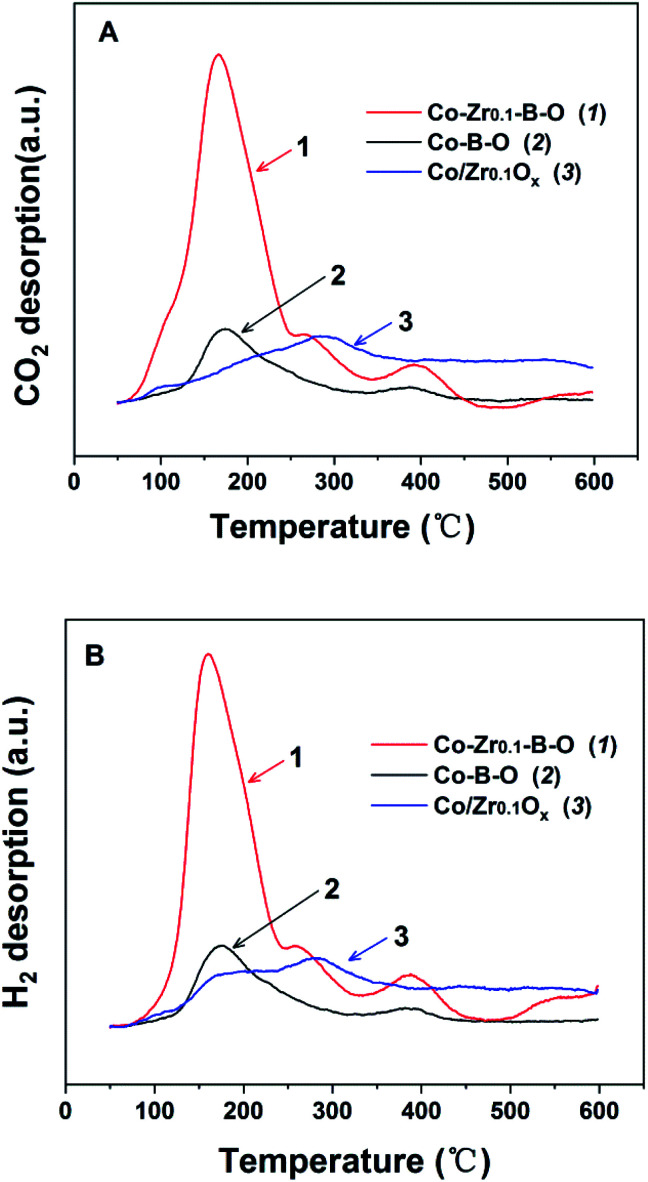
The TPD profiles of Co–B–O, Co–Zr_0.1_–B–O and Co/Zr_0.1_O_*x*_ catalysts: (A) CO_2_-TPD, (B) H_2_-TPD. The TPD signals have been normalized to the mass of the tested samples.

To understand the impact of the peculiar structure on the reaction, we conducted the temperature programmed desorption (TPD) analysis, *i.e.*, CO_2_-TPD and H_2_-TPD (Fig. 6). The results demonstrated that the major desorption peaks of CO_2_ and H_2_ appeared at closely below 180 °C, and the peaks of the Co–Zr_0.1_–B–O catalyst were significantly larger than those of the Co–B–O catalyst. This may account for the better performance of the Co–Zr_0.1_–B–O catalyst than the Co–B–O catalyst. The peaks of CO_2_ and H_2_ of the Co/Zr_0.1_O_*x*_ catalyst were observed at much higher temperature (nearly 300 °C), and were much smaller than those of the Co–Zr_0.1_–B–O catalyst. This also helps to explain why the catalytic activity of the amorphous Co–Zr_0.1_–B–O catalyst was markedly higher than that of the Co/Zr_0.1_O_*x*_ catalyst fabricated by the commonly reported methods.

In most cases of CO_2_ methanation, the undesired RWGS reaction also occurred, especially at relatively high temperature (>300 °C). The reaction route of CO_2_ methanation also depends on the composition and structure of the catalyst.^42,43^ In this work, no CO was observed under all conditions. Moreover, CO hydrogenation could hardly take place over the Co–Zr_0.1_–B–O catalyst, and the very small amount of CO consumed in the reaction was mostly converted to CO_2_ (Table S4†). This suggested that the CO disproportionation (CO → CO_2_ + C) occurred in the reaction. The carbon deposit generated *in situ* blocked the active sites and inhibited further reaction.^44^ To study the impact of the reactants on the valence of the catalyst, we conducted XPS characterization of the Co–Zr_0.1_–B–O catalysts after H_2_ adsorption and subsequent CO_2_ adsorption at reaction temperature (Fig. S12†). The results revealed that under the reaction conditions Co^0^ and Co^2+^ coexisted on the catalyst surface and the ratio of Co^0^/Co^2+^ fluctuated with the sequential introduction of H_2_ and CO_2_. Moreover, the valencies of Zr, B, O fluctuated synchronously with that of Co. The synergy of these elements in the catalytic cycles also helps to explain the excellent catalytic performance.

To detect the intermediates of the reaction, we conducted the *in situ* Fourier transform infrared (FTIR) analysis of CO_2_ adsorption on the catalyst pretreated with H_2_ at 180 °C (Fig. S13†). The result revealed that CO_2_ was quickly reduced by the surface H atoms into intermediates, such as formic acid (HCOOH, 1080) and methoxyl (CH_3_O, 1051 cm^−1^) adsorbed on the catalyst surface.^45^ Several remarkable peaks between 2000 and 2150 cm^−1^ were also observed, which could be ascribed to the intermediates H_*x*_CO_*y*_ (*x* = 1–2; *y* = 2–3) formed by H_2_, CO_2_ and/or hydroxyl groups on the catalyst surface.^46^ All these intermediates adsorbed weakly on the catalyst surface and disappeared immediately when the CO_2_ flow was stopped and H_2_ was introduced again. The *in situ* FTIR spectrum of the catalyst with simultaneous introduction of CO_2_ and H_2_ is given in Fig. S14.† The peaks of the above intermediates became very small or could not be observed at all, which also suggested their quick transformation under CO_2_ methanation conditions. The major peaks (1210, 1330 and 1573 cm^−1^) are ascribed to the intrinsic feature of the catalyst evolved at the reaction temperature (Fig. S15†). The quick formation and conversion of the reactive intermediates at the reaction temperature may account for the very high activity of the catalyst. Some intermediates (HCO_3_^−^, –COO^−^, and CH_3_O–) were also observed by XPS characterization of the catalyst after CO_2_ methanation (Fig. S16†). These intermediates may be formed and preserved during cooling of the reactor.

Based on above discussion, we proposed the possible reaction mechanism. The CO_2_ and H_2_ adsorbed on the surface of the Co–Zr_0.1_–B–O catalyst, where the Zr promoter greatly and simultaneously enhanced their adsorption capabilities. The CO_2_ and H_2_ reacted on the catalyst surface, where the Co, Zr, B and O atoms worked cooperatively. The CO_2_ was reduced by the H atoms to methane *via* a series of intermediates, such as HCO_3_^−^, HCOO^−^, HCOOH, CH_3_O–, and other possible species H_*x*_CO_*y*_ (*x* = 1–2; *y* = 2–3) containing carbonyl. Under optimized conditions, reactive intermediates were generated and transformed very quickly, accounting for the very high catalytic activity. The outstanding performance of the catalyst may originate from its strong ability to adsorb both reactants and the synergy of the atoms on the catalyst surface in converting them.

## Conclusions

In summary, we report an amorphous Co–Zr_0.1_–B–O catalyst for CO_2_ methanation. The catalyst was very active and selective, and the activity of the catalyst reached 10.7 mmol_CO_2__ g_cat_^−1^ h^−1^ at 180 °C with a methane selectivity of 97.8%. The promoting factor of the Co–Zr_0.1_–B–O catalyst was as high as 12.3, which is remarkably higher than those of the reported catalysts. It is noteworthy that the catalytic performance is comparable to or even higher than that of some noble metal catalysts under similar conditions. The outstanding performance of the catalyst originated from two aspects. Firstly, the amorphous nature of the catalyst may lead to abundant surface defects and intrinsic active sites. Secondly, the Zr promoter could increase the surface area of the catalyst, enrich the Co atoms on the catalyst surface, and tune the valence state of the atoms at the catalyst surface. All these factors may enhance the activity of the catalyst. In the reaction, CO_2_ was reduced by the H atoms to methane *via* a series of intermediates, such as HCO_3_^−^, HCOO^−^, HCOOH, CH_3_O^−^, and other possible species H_*x*_CO_*y*_ (*x* = 1–2; *y* = 2–3) containing carbonyl. We believe that this cheap and highly efficient catalyst has promising potential applications, and the protocol to design amorphous catalysts with promoters is useful to prepare other efficient catalysts using cheap metals.

## Experimental

### Chemicals

Sodium borohydride (98.0%), cobalt acetate tetrahydrate (99.5%), zinc nitrate hexahydrate (99.0%), CO_2_ (≥99.99%) and H_2_ (≥99.99%) were purchased from Sinopharm Chemical Reagent Co., Ltd (SCR). Zirconium nitrate pentahydrate (98%+) was provided by Adamas Reagent, Ltd. Cerium(iii) nitrate hexahydrate (99.5%) was offered by J&K, aluminum nitrate nonahydrate (99.99%) and chromium(iii) nitrate nonahydrate (99.95%) were bought from Aladdin.

### Catalyst preparation

Metal-doped Co–B–O catalysts were prepared through the liquid phase reduction. Typically, solution A was first prepared by dissolving 0.303 g sodium borohydride in 10 mL distilled water containing 0.5 mL ammonia. 2 mmol cobalt acetate tetrahydrate and 0.2 mmol metal salts (M = Ce, Al, Zr, Cr, Zn) were dissolved in a mixed solution of 20 mL distilled water and 5 mL ethanol to prepare solution B. Then solution A was added into solution B quickly at room temperature under vigorous stirring. The mixture was kept stirring for 20 min. Then the catalyst denoted as Co–M_0.1_–B–O was obtained after centrifuging, washing with distilled water (200 mL), ethanol (30 mL) and acetone (30 mL), drying overnight under vacuum at 80 °C. Co–Zr_*x*_–B–O with different Zr amounts (*X* = *n*_Zr_/*n*_Co_, *X* = 0.05, 0.3) was synthesized by a similar method. Zr–B–O and Co–B–O were synthesized using the above steps except for the absence of cobalt acetate tetrahydrate and zirconium nitrate, respectively.

Co/Zr_0.1_O_*x*_ was synthesized by a coprecipitation method. 2 mmol cobalt acetate tetrahydrate and 0.2 mmol zirconium nitrate were dissolved in a mixed solution of 20 mL distilled water and 5 mL ethanol, then 10 mL of sodium hydroxide solution (0.8 mol mL^−1^) was added dropwise into the above solution under stirring, and after stirring for another 20 min a dark blue precipitate was obtained by filtration, followed by washing with 200 mL distilled water and drying overnight at 80 °C. The Co/Zr_0.1_O_*x*_ was obtained by calcination of the precipitate in air at 400 °C for 3 h followed by reduction with 5% H_2_ in Ar at 400 °C for 1 h.

### Catalyst characterization

The N_2_ adsorption–desorption isotherms were recorded at 77 K using an ASAP2020 (Micromeritics, USA). The catalysts were treated under vacuum at 150 °C for 4 h before the test. The specific surface area was obtained by the Brunauer–Emmett–Teller (BET) method. XRD patterns of the catalysts were obtained using a Rigaku Ultima IV X-ray diffractometer with Cu Kα radiation (*λ* = 1.5418 Å) at a rate of 20.0° min^−1^ over the range of 20–90°. The X-ray photoelectron spectroscopy (XPS) experiment was conducted on an AXIS SUPRA (Kratos Corp.) with an Al Kα excitation source and all binding energies were referenced to the C 1s at 284.8 eV. High resolution transmission electron microscopy (HRTEM) was conducted on a Tecnai G2 F30 FETEM (FEI Corp.). HAADF-STEM images and EDS mapping were obtained using a Tecnai G2 F20 (FEI Corp). FT-IR spectra were collected using an NEXUS670 Fourier transform infrared spectrometer (ScanStantion C5, USA).

CO_2_-temperature programmed desorption (TPD) was conducted using a Micromeritics AutoChem II chemisorption analyzer with He (30 mL min^−1^) as the carrier gas. About 0.1 g catalyst was charged into the quartz tube and heated to 100 °C at the rate of 20 °C min^−1^. After 1 h the temperature was cooled down to 50 °C, and CO_2_ adsorption proceeded with 10% CO_2_–90% He (v/v) mixed gas for 30 min with a flow rate of 50 mL min^−1^. Then the sample was purged with He of 50 mL min^−1^ for 1 h. Finally, CO_2_ desorption proceeded from 50 to 600 °C at 10 °C min^−1^. The H_2_-TPD measurement was performed using the same equipment with a similar procedure, except that 10% H_2_–90% He (v/v) was used.

*In situ* FTIR spectra were recorded with a NICOLET iS50 FT-IR spectrometer (Thermo SCIENTIFIC, USA) equipped with a high-temperature reaction chamber and a mercury cadmium telluride (MCT) detector at a resolution of 4 cm^−1^ and 32 scans per spectrum. The Co–Zr_0.1_–B–O catalyst and KBr were ground together and put into the sample cup. Then two tests were carried out using fresh catalyst, respectively. The first test was as follows: at 180 °C, the sample was first purged with N_2_ (100 mL min^−1^) for 2 h, and then it was treated with H_2_ (50 mL min^−1^) for 2 h. After further purging the sample with N_2_ (100 mL min^−1^) for 1 h, CO_2_ (100 mL min^−1^) was introduced and the CO_2_ adsorption began. Then the sample was further purged with N_2_ (100 mL min^−1^) for 2 h. In the end, it was treated with H_2_ (50 mL min^−1^) for 3 h. The background spectrum was recorded before CO_2_ adsorption. The second test was as follows: the chamber was first purged at 180 °C with N_2_ for 2 h and cooled down to 20 °C. After the CO_2_ was introduced for 5 min, both CO_2_ and H_2_ were charged and the sample was heated from 20 to 180 °C at the rate of 5 °C min^−1^ and kept at 180 °C for 0.5 h. The background spectrum was scanned before CO_2_ was introduced.

### Catalytic reaction

The methanation of CO_2_ was conducted in a 16 mL stainless-steel autoclave. Typically, 40 mg catalyst and 2 mL cyclohexane were added in the reactor. The reactor was sealed and the air in it was substituted with CO_2_ of 1 MPa three times, and then 4 MPa CO_2_ and 4 MPa H_2_ were charged successively at room temperature. The reactor was heated to 180 °C under stirring and was kept for 12 h. After the reaction, the reactor was cooled in an ice-water bath, and the residual gas was released slowly and collected for GC analysis (Agilent 7890A) equipped with a thermal conductivity detector (TCD) and flame ionization detector (FID). The amount of hydrocarbons such as methane was determined on the FID using a HP-AL/S column. The amount of carbon dioxide and hydrogen was obtained on the TCD using a HP-PLOT/Q column. The liquid phase was analyzed using an Agilent Technologies 7890B GC system with a flame ionization detector using a HP-5 column. The conversion of CO_2_ was the percentage of the CO_2_ charged into the reactor that was converted to hydrocarbon products, as is given below.
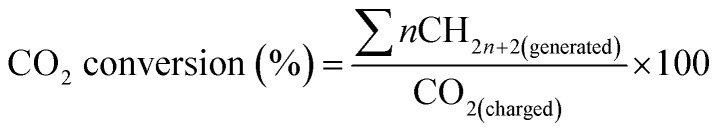


Because H_2_ was the limiting reactant and it could more effectively reflect the proceeding of the reaction, calculation of the methane yield was based on the H_2_ charged into the reactor, as is shown below.
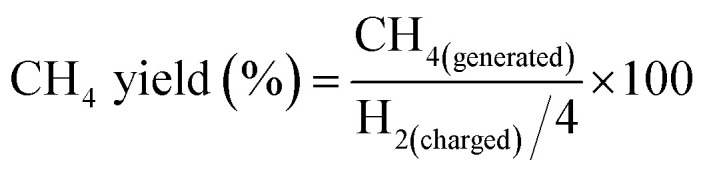


The CH_4_ selectivity was the percentage of the C atoms in CH_4_ over the C atoms in total products, as is given below.
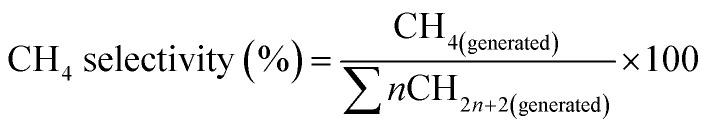


### The recycling test

After the reaction, the residual gas was released slowly. The gaseous and liquid samples were analyzed respectively. Then the reactor was sealed and fresh reactants (CO_2_ and H_2_) were charged to start the next run.

## Conflicts of interest

There are no conflicts to declare.

## Supplementary Material

SC-012-D0SC06414A-s001
