# Supplementary material for: Low temperature methanation of CO2 over an amorphous cobalt-based catalyst
Source: Chem Sci. 2021 Jan 15;12(11):3937–43. doi: 10.1039/d0sc06414a (PMC8179427; doi:10.1039/d0sc06414a)
Supplement: SC-012-D0SC06414A-s001 [file SC-012-D0SC06414A-s001.pdf]

Electronic Supplementary Material (ESI) for Chemical Science.
This journal is © The Royal Society of Chemistry 2021

Supplementary Information

Low Temperature Methanation of CO_2 over an Amorphous Cobalt-based Catalyst

Jinghui Tu,[a] Haihong Wu,[a]* Qingli Qian,[b]* Shitao Han,[a] Mengen Chu,[a] Shuaiqiang Jia,[a] Ruting Feng, [a] Jianxin Zhai,[a] Mingyuan He,[a] Buxing Han [a,b]*

[a] Shanghai Key Laboratory of Green Chemistry and Chemical Processes, School of Chemistry and Molecular Engineering, East China Normal University, Shanghai 200062, P. R. China

[b] Beijing National Laboratory for Molecular Sciences, CAS Key Laboratory of Colloid and Interface and Thermodynamics, CAS Research/Education Center for Excellence in Molecular Sciences, Institute of Chemistry, Chinese Academy of Sciences, China

E-mail: hhwu@chem.ecnu.edu.cn; qianql@iccas.ac.cn; hanbx@iccas.ac.cn

Figures and Tables

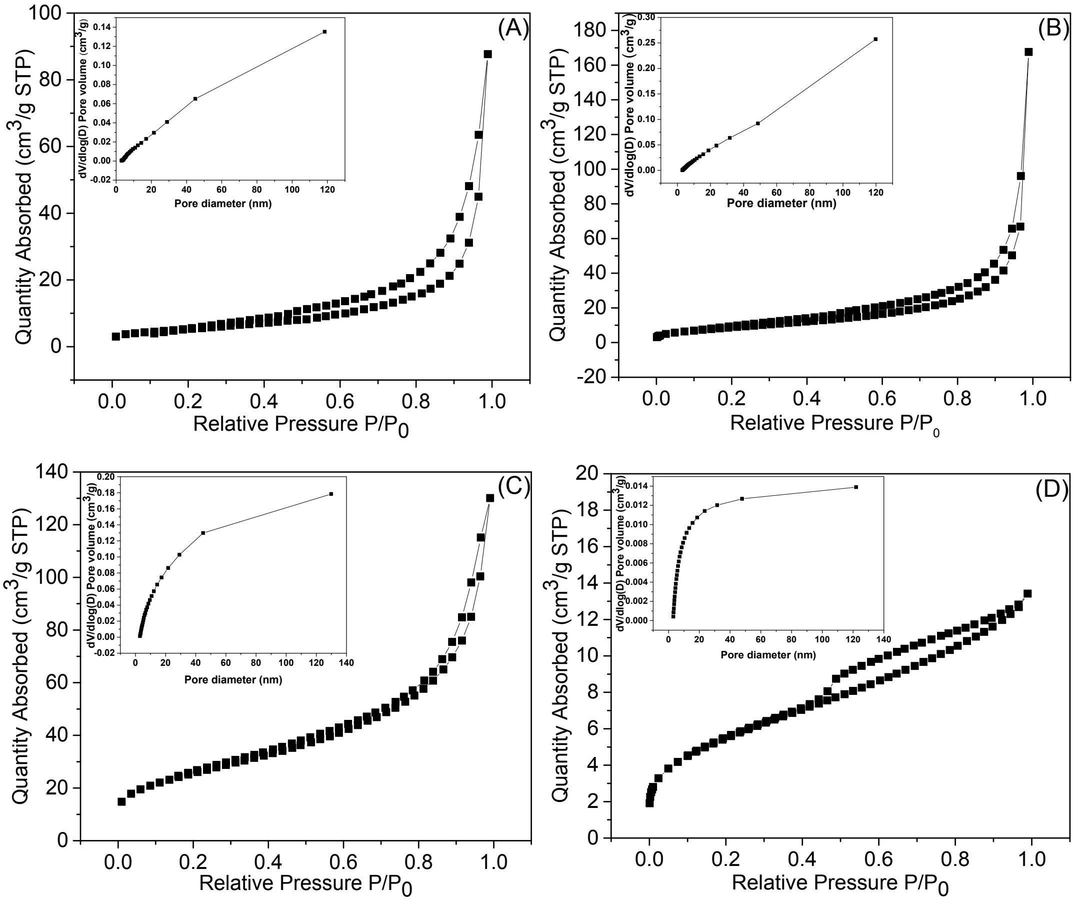

Figure S1. The N_2 adsorption isotherm and pore diameter distribution for the catalysts (A) Co-B-O, (B) Co-$Zr_{0.05}$-B-O, (C) Co-$Zr_{0.1}$-B-O, (D) Co-$Zr_{0.3}$-B-O.

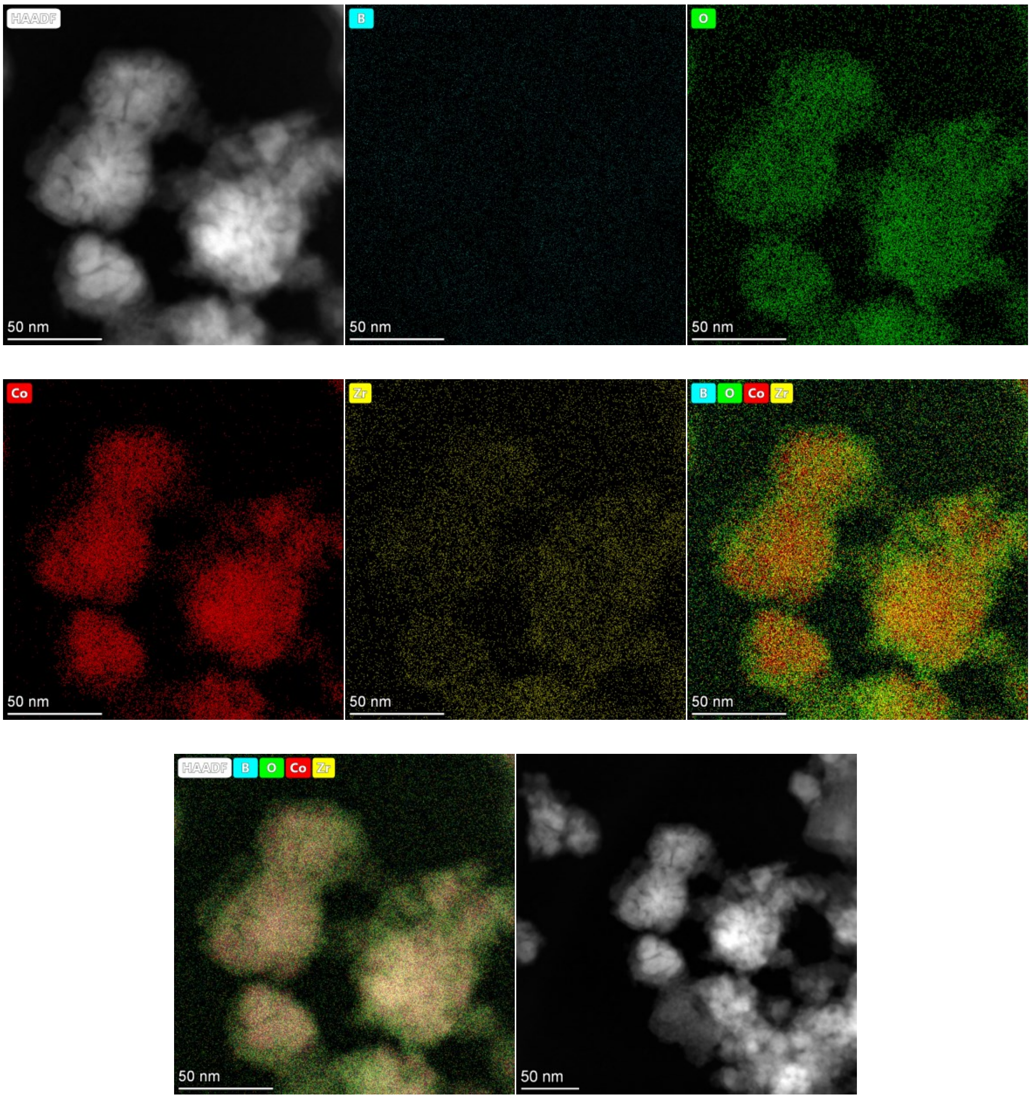

Figure S2. The HAADF-STEM images and EDS mapping of B, O, Co and Zr on the Co-$Zr_{0.1}$-B-O catalyst.

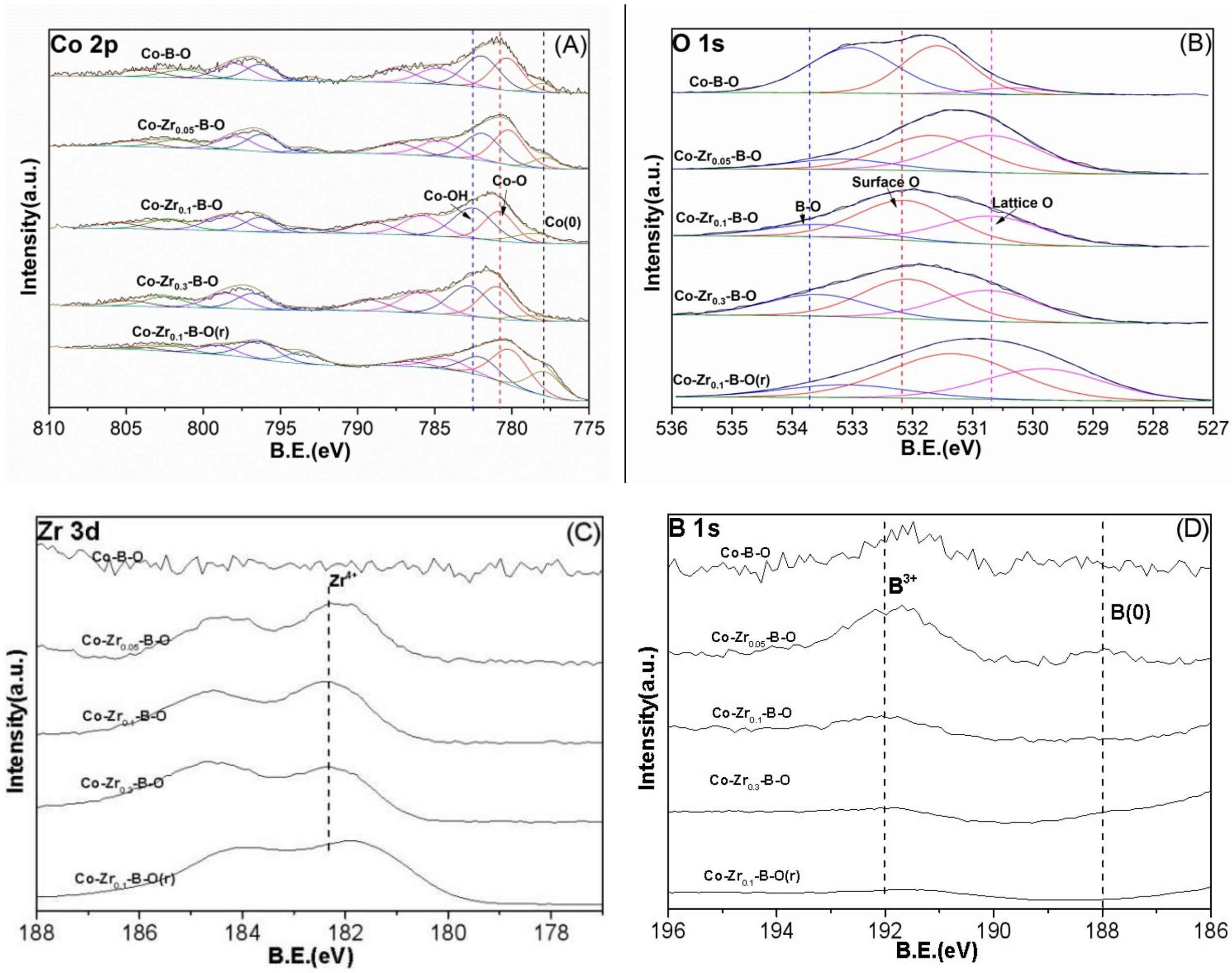

Figure S3 XPS spectra of the Co-Zr_x-B-O catalysts: (A) Co2p; (B) O1s; (C) Zr3d; (D) B1s. The data were fitted according to literature.[1]

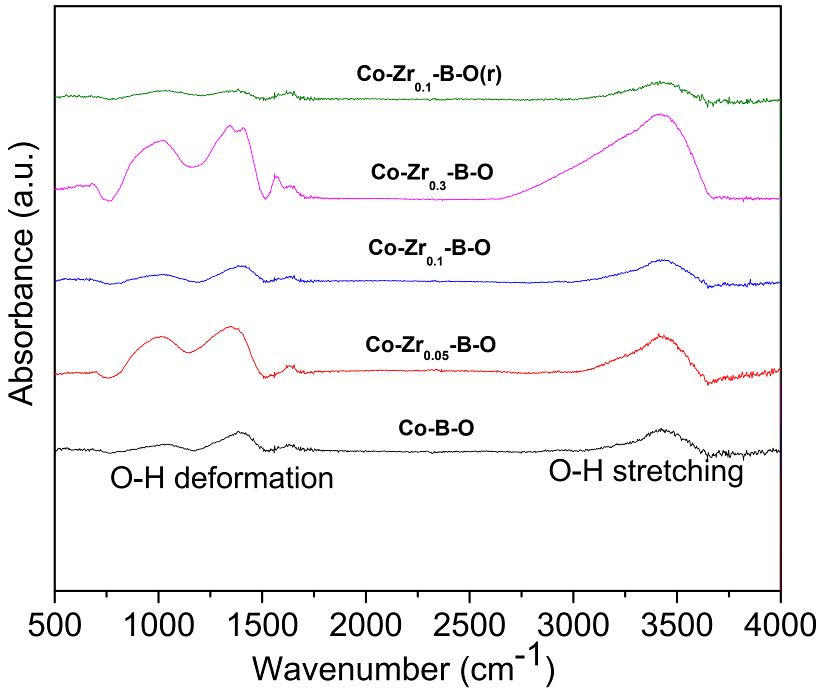

Figure S4. The FTIR spectra of the Co-$Zr_{0.1}$-B-O catalysts. The peak positions of O-H deformation and stretching are the same as those in the literature.[2]

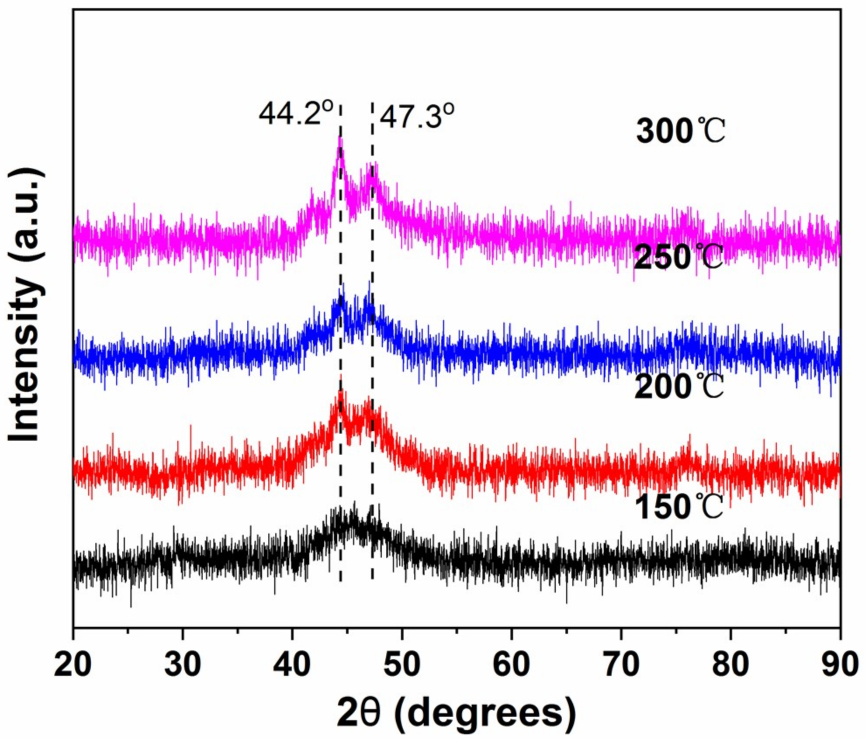

Figure S5. The XRD spectra of the Co-$Zr_{0.1}$-B-O catalyst after treating in flowing H_2 at different temperature.

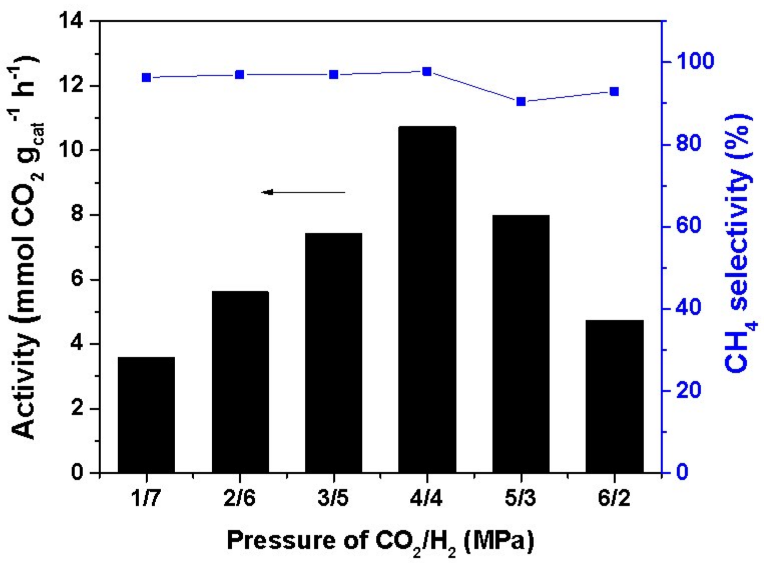

Figure S6. Impact of P_{CO2}/P_{H2} ratio upon the reaction. Conditions: Co-$Zr_{0.1}$-B-O catalyst 40 mg, cyclohexane 2 mL, initial pressure 8.0 MPa, 180 ^oC, 12 h.

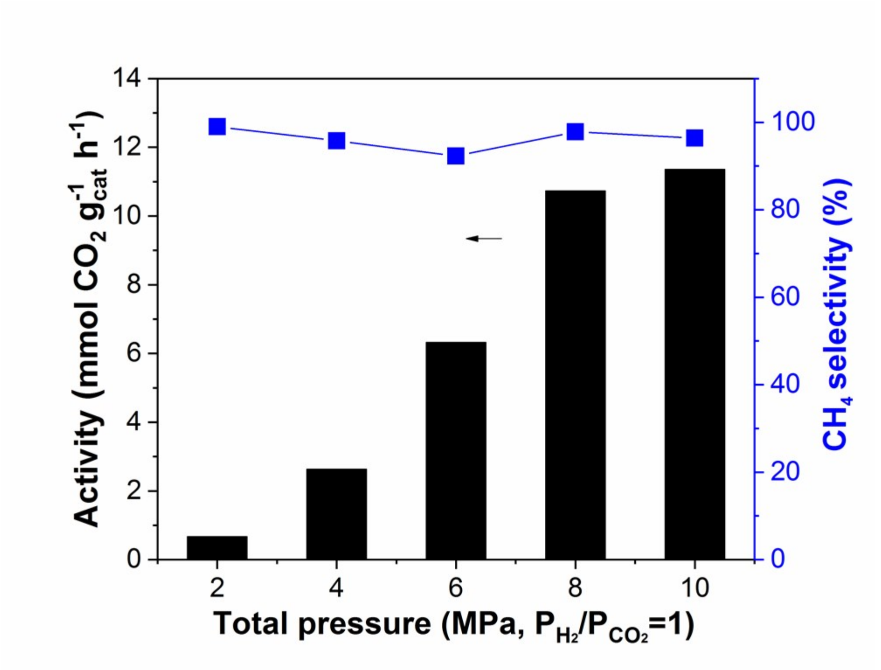

Figure S7. Impact of total pressure on the reaction. Conditions: Co-$Zr_{0.1}$-B-O catalyst 40 mg, cyclohexane 2 mL, P_{CO2}/P_{H2}=1, 180 ^{o}C, 12 h.

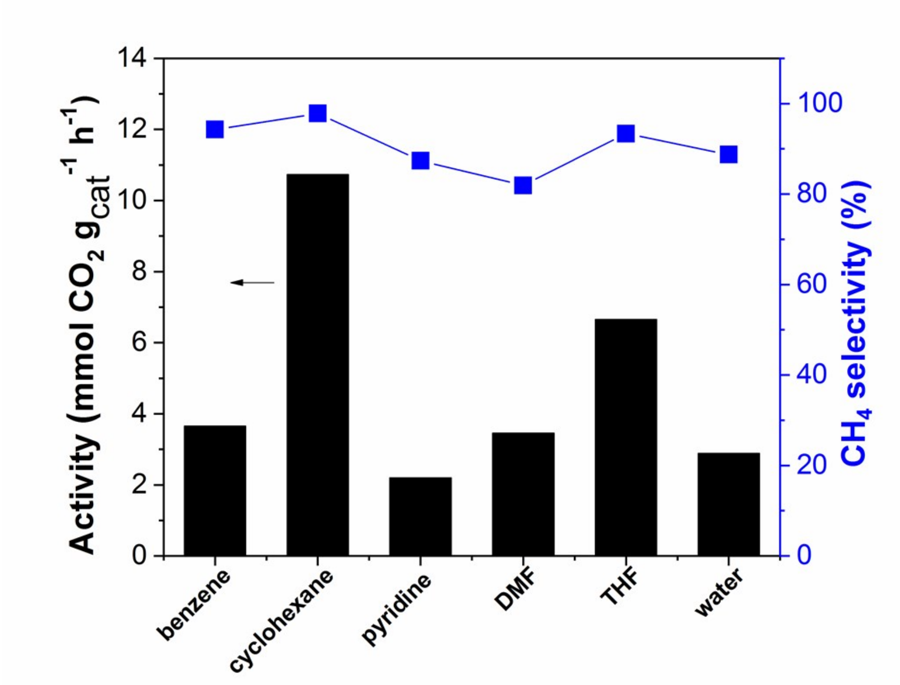

Figure S8. The results of the reaction in different solvents. Conditions: Co-$Zr_{0.1}$-B-O catalyst 40 mg, solvent 2 mL, 4 MPa CO_2, 4 MPa H_2, 180 ^oC, 12 h.

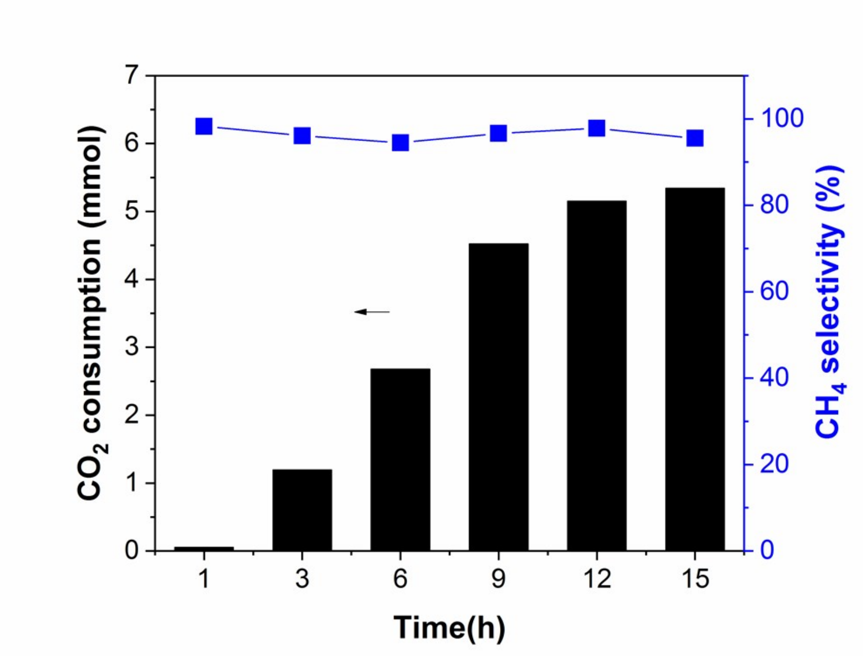

Figure S9. Time course of the reaction. Conditions: Co-$Zr_{0.1}$-B-O catalyst 40 mg, cyclohexane 2 mL, 4 MPa CO_2, 4 MPa H_2, 180 ^oC.

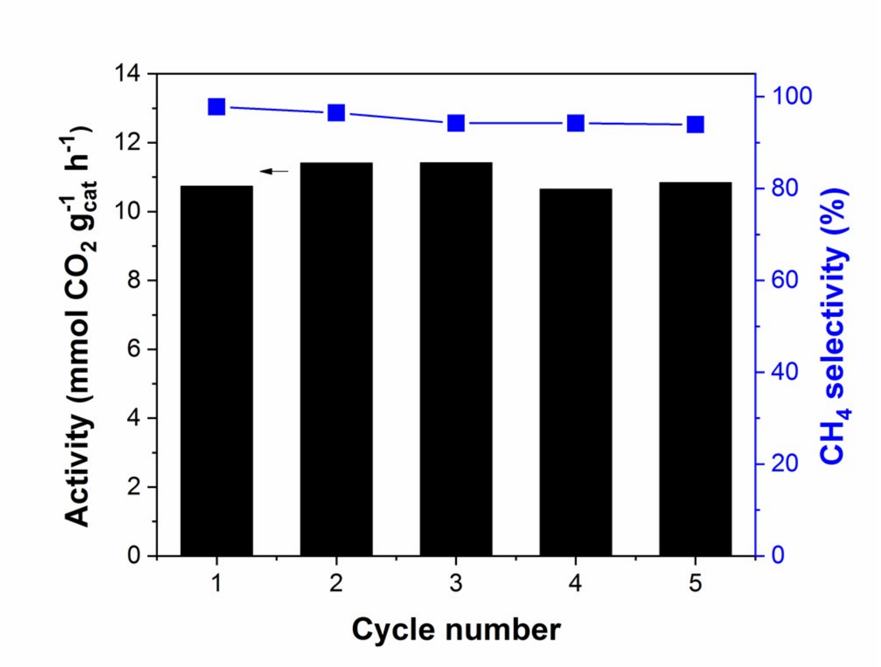

Figure S10. Results of the recycling test of the catalyst. Conditions: Co-$Zr_{0.1}$-B-O catalyst 40 mg, cyclohexane 2 mL, 4 MPa CO_2, 4 MPa H_2, 180 ^oC, 12 h.

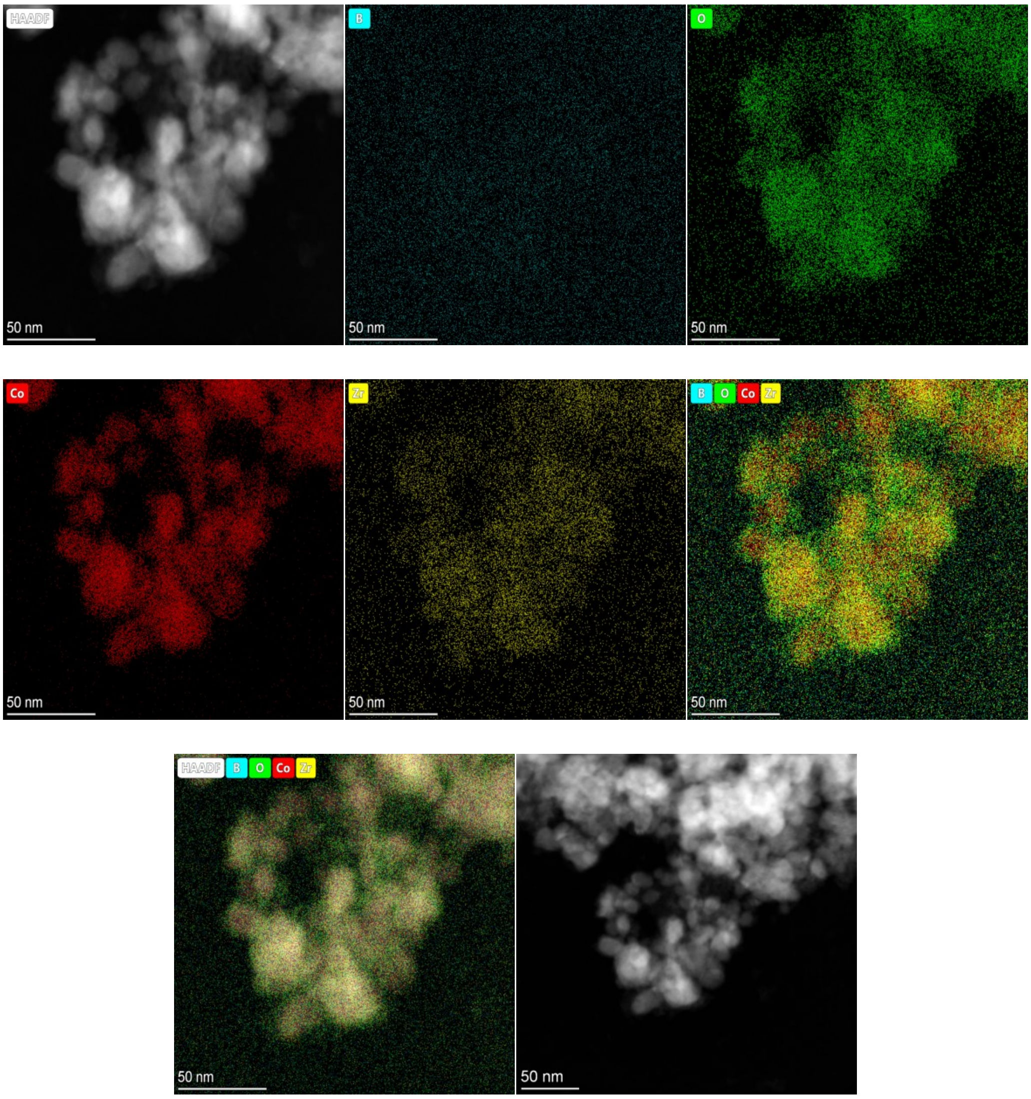

Figure S11. The HAADF-STEM images and EDS mapping of B, O, Co and Zr on the catalyst after five cycles.

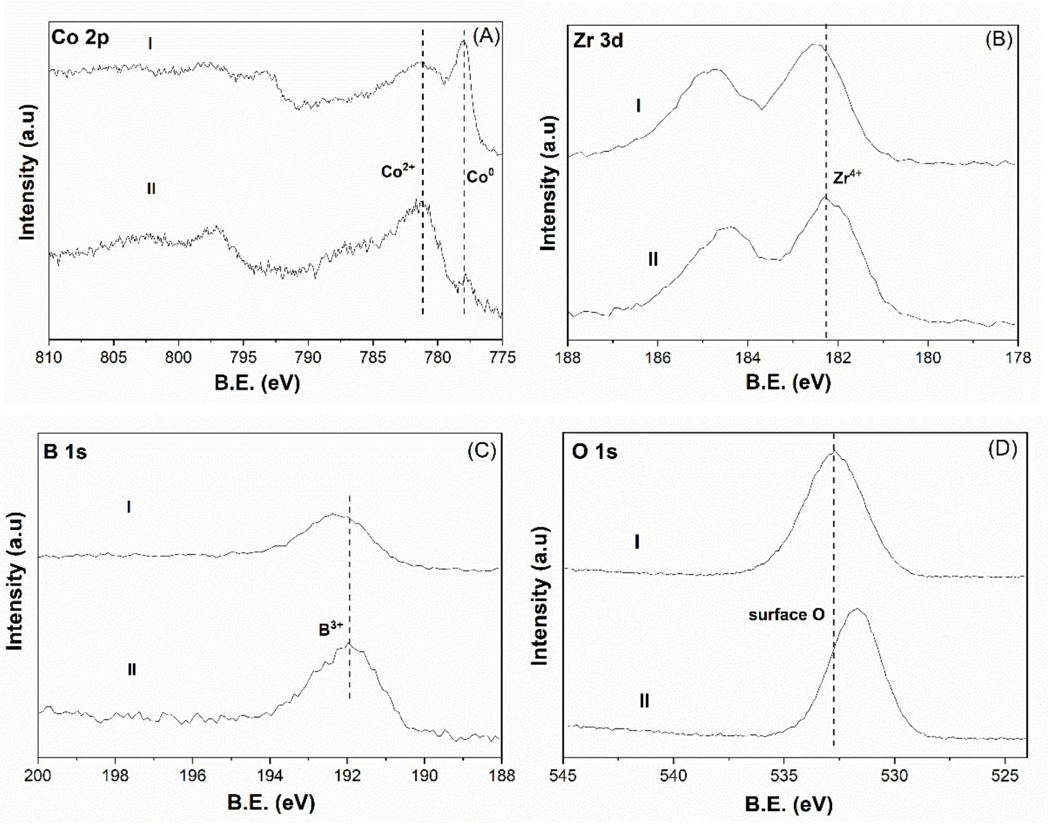

Figure S12. XPS characterization of the Co-$Zr_{0.1}$-B-O catalyst after adsorption of H_2 or CO_2 at elevated temperature: (A) Co 2p, (B) Zr 3d, (C) B 1s, (D) O1s.

I: The catalyst was pretreated in a reactor with 4 MPa H_2 at 180 ^oC for 2 h and then cooled to room temperature;

II: After step I the H_2 was released and replaced by 4 MPa CO_2, after the reactor was kept at 180 ^oC for 1 h it was cooled to room temperature.

After the adsorption, the catalyst samples in the reactors were quickly transferred into the chamber of the XPS spectrometer.

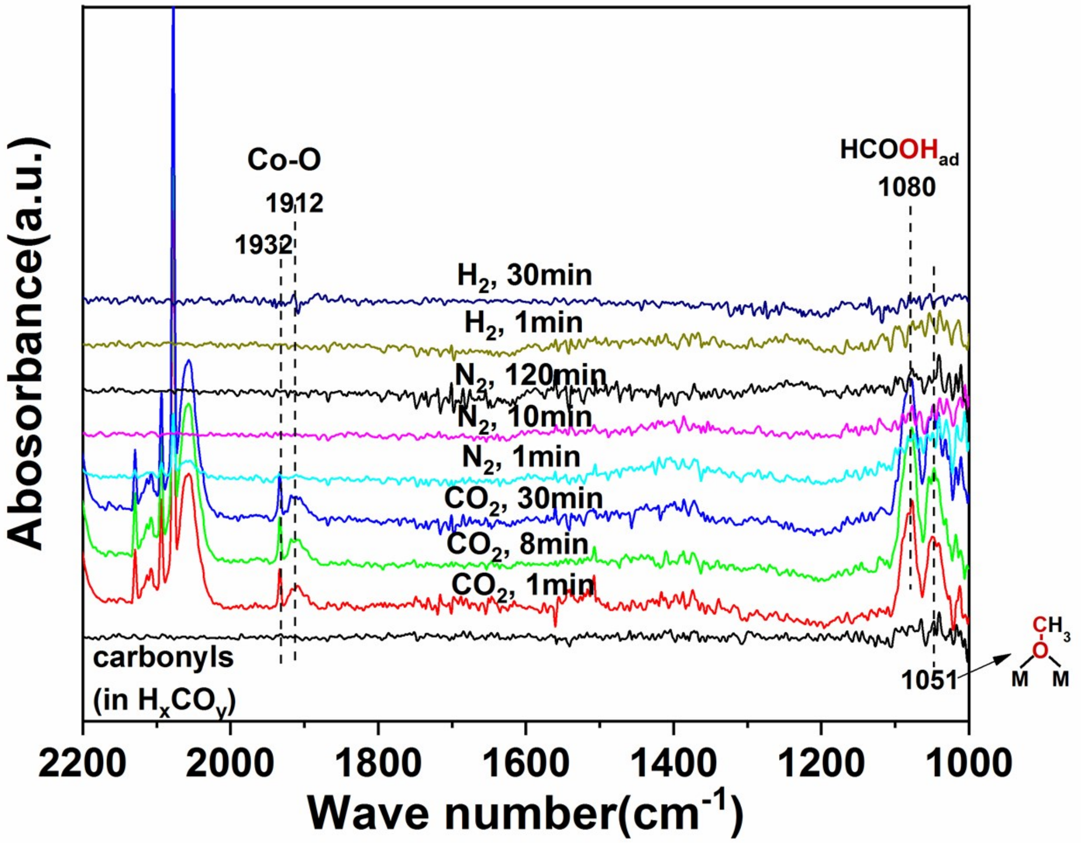

Figure S13. In situ FTIR spectra obtained during CO_2 adsorption on the Co-$Zr_{0.1}$-B-O catalyst. The catalyst was pretreated by H_2 at 180 ^oC for 2 h before the CO_2 adsorption. The black line is the background spectrum scanned before CO_2 was introduced.

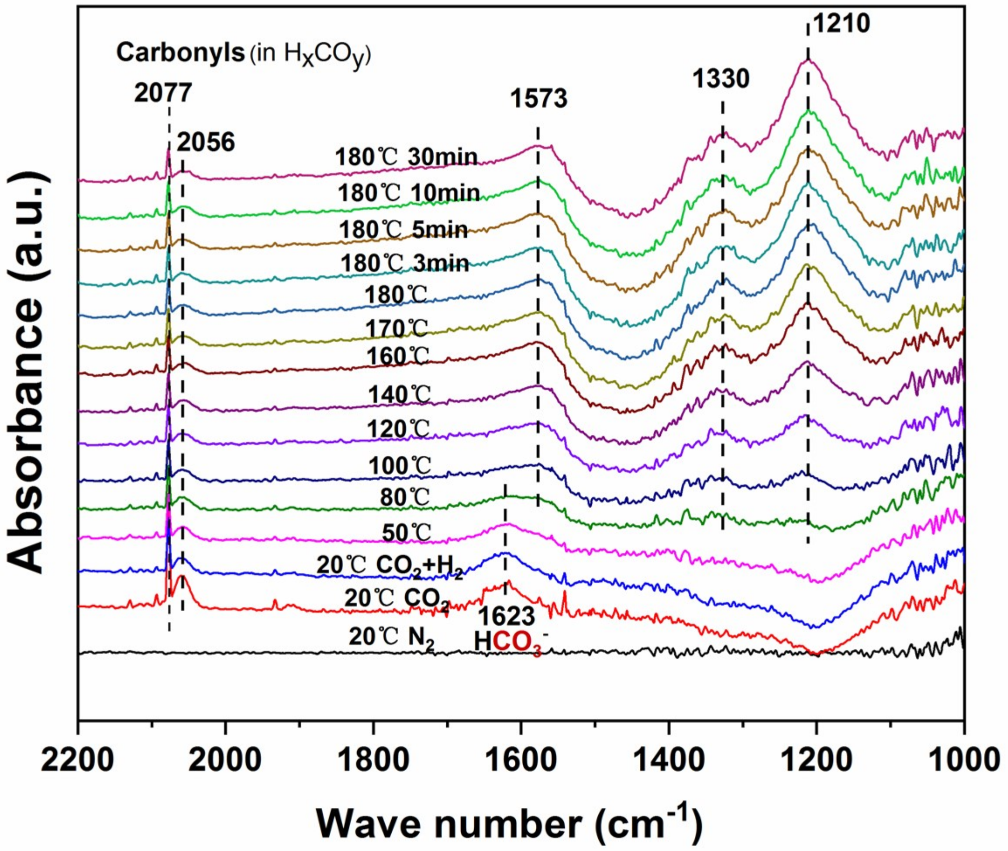

Figure S14. In situ FTIR spectra during reaction of CO_2 and H_2 over the Co-$Zr_{0.1}$-B-O catalyst. At 20 ^oC, CO_2 was firstly added, then CO_2 and H_2 were introduced together. The temperature was elevated from 20 to 180 ^oC at the rate of 5 ^oC /min and kept at 180 ^oC for 30 min. The black line is the background spectrum scanned at 20 ^oC before CO_2 was introduced.

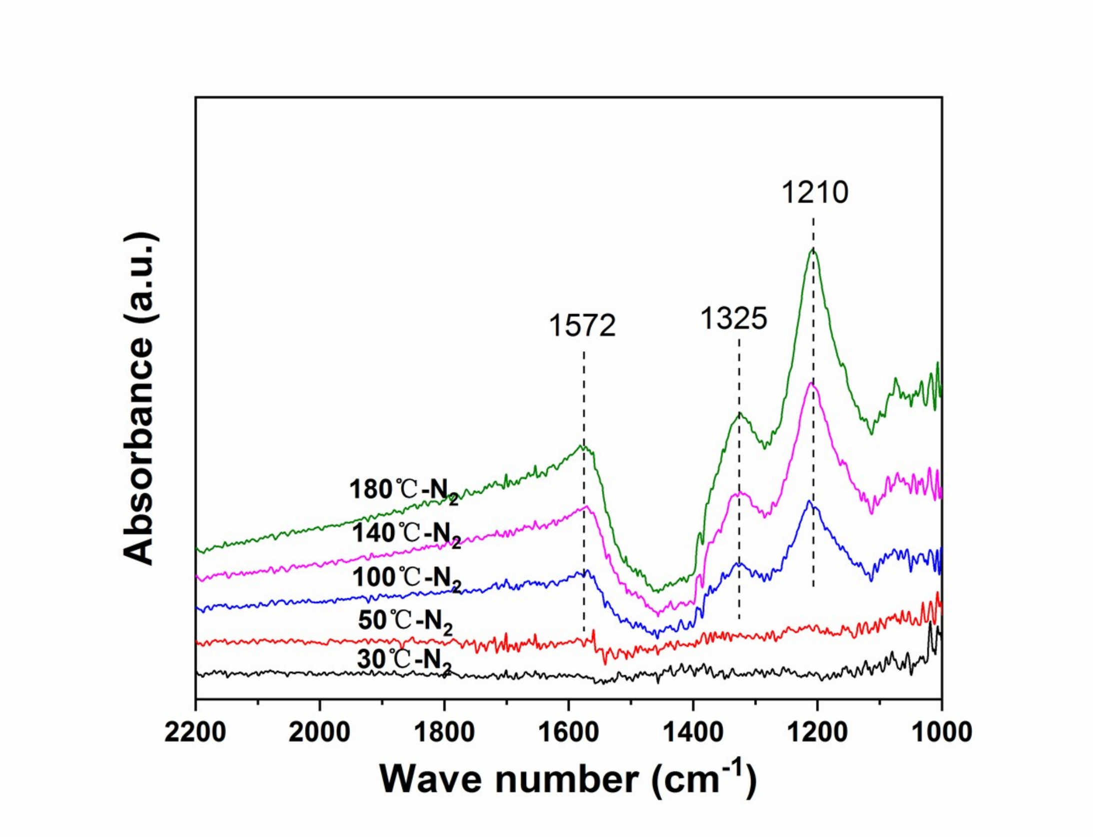

Figure S15. In situ FTIR spectra of the Co-$Zr_{0.1}$-B-O catalyst during heating at a rate of 10 ^oC/min in N_2 flow. The background spectrum was scanned at 30 ^oC.

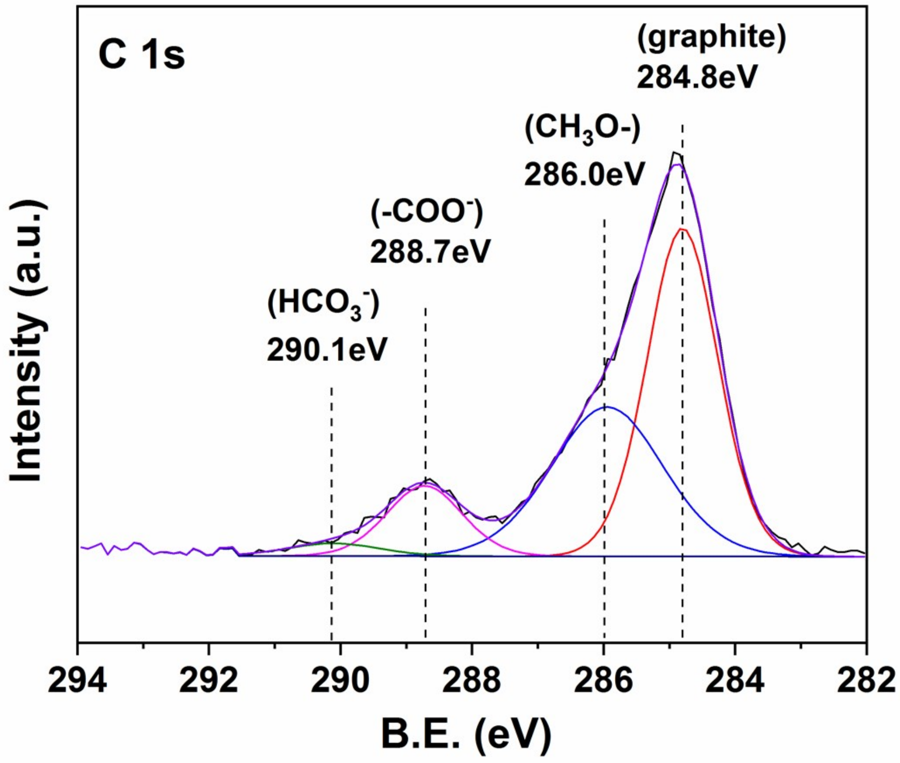

Figure S16. XPS characterization of the Co-$Zr_{0.1}$-B-O catalyst after CO_2 methanation. Conditions: Co-$Zr_{0.1}$-B-O catalyst 40 mg, cyclohexane 2 mL, 4 MPa CO_2, 4 MPa H_2, 180 ^oC, 12 h. The sample was obtained after releasing the gases of the cooled reactor.

Table S1. The representative results of CO_2 methanation at low temperature (≤250 ^oC).

Entry	Catalyst	*T* (^oC)	Best data in the Reference	Normalized activity ($mmol_{CO2}\cdot g_{cat}^{-1}h^{-1}$)	CH_4%	Ref.
1	Ru(NC)/CeO_2	190	7.41×10^{-3} s^{-1}	6.8	>98	[3]
2	Rh(3 wt.%)/TiO_2	150	0.143×10^{-2} mol_{CH4} mol_{Rh}^{-1} s^{-1}	1.5	100	[4]
3	Ru/[BMIM]BF_4/SiO_2	250	70% (CO_2 conversion)	5.1	100	[5]
4	RuNPs in [C_8mim][NfO]	150	3.25 h^{-1} (84% yield)	n/a	100	[6]
5	Ru/TiO_2 (B)	160	1.5×10^{-2} S^{-1}	2.4	100	[7]
6	RuNPs in [omim][NTf_2]	150	72 (TON), 69% (yield)	29.7	100	[8]
7	Rh-Pd/γ-Al_2O_3	200	0.309 umol g_{cat}^{-1} s^{-1} (0.318×10^{-2} mol_{CH4} mol_{Rh}^{-1} s^{-1})	1.1	100	[9]
8	3Ru-30Ni/$Ce_{0.9}Zr_{0.1}O_2$	230	98.2% (CO_2 conversion)	21.0	100	[10]
9	Ru/30%CeO_2/Al_2O_3	250	6.2 mL min^{-1} g_{cat}^{-1}	16.6	≈100	[11]
10	Rh/γ-Al_2O_3:Ni/AC	125	9.5 umol g^{-1}	n/a	100	[12]
11	Co-$Zr_{0.1}$-B-O	180		10.7	97.8	This work

The activities of some results are converted to $mmol_{CO2}$ • $g_{cat}^{-1}h^{-1}$ based on the data given in the literatures, and the detailed procedures are given below.

n/a represents "not available" because of lacking some key parameter for calculation.

Ru(NC)/CeO_2 (Ref. 3):

7.41×10^{-3} s^{-1} × 3600 s/h ÷ 101.1 g/mol_{Ru} × 0.0256 g_{Ru}/g_{cat} × 1000 mmol/mol = 6.8 $mmol_{CO2}\cdot g_{cat}^{-1}h^{-1}$

Rh(3 wt.%)/TiO_2 (Ref. 4):

0.143×10^{-2} mol_{CH4} $molRh^{-1}$ s^{-1} × 3600 s/h ÷ 102.9 g/mol_{Rh} × 0.03 g_{Rh}/g_{cat} × 1000 mmol/mol = 1.5 $mmol_{CO2}\cdot g_{cat}^{-1}h^{-1}$

Ru/[BMIM]BF_4/SiO_2 (Ref. 5):

2400 h^{-1} (GHSV) × l L_{cat} ÷ 22.4 L/mol × 15 (CO_2)% × 70 (conv.)% ÷ (1 L_{cat} × 2200 g_{cat}/L) × 1000 mmol/mol = 5.1 $mmol_{CO2}\cdot g_{cat}^{-1}h^{-1}$

(The catalyst density was not available and was supposed to be the density of SiO_2 support because of the low Ru loading.)

Ru/TiO_2 (B) (Ref. 7):

1.5×10^{-2} s^{-1} × 1.55 $m^2g_{cat.}^{-1}$ × 0.1739 $\times10^{20}$ ÷ (6.02×10^{23} atom/mol) × 1000 mmol/mol × 3600 s/h = 2.4 $mmol_{CO2}\cdot g_{cat}^{-1}h^{-1}$

RuNPs in [omim][NTf_2] (Ref. 8):

72 mol_{CH4} mol_{Ru}^{-1} ÷ 101.1 g/$mo1_{Ru}$ ÷ 24h × 1000 mmol/mol = 29.7 $mmol_{CO2}\cdot g_{cat}^{-1}h^{-1}$

Rh-Pd/γ-Al_2O_3 (Ref. 9):

0.309 umol g_{cat}^{-1} s^{-1} × 3600 s/h ÷ 1000 umol/mmol = 1.1 $mmol_{CO2}\cdot g_{cat}^{-1}h^{-1}$

(The reaction rate with the unit of umol g_{cat}^{-1} s^{-1} was obtained directly from Table 1 of the Ref. 9)

3Ru-30Ni/$Ce_{0.9}Zr_{0.1}O_2$ (Ref. 10):

2400 mL $g^{-1}h^{-1}$ ÷ 22.4 mL/mmol × $20\%_{CO2}$ × $98.2\%_{conv.}$ = 21.0 $mmol_{CO2}\cdot g_{cat}^{-1}h^{-1}$

Ru/30%CeO_2/Al_2O_3 (Ref. 11):

6.2 mL min^{-1} g_{cat}^{-1} ÷ 22.4 mL/mmol × 60 min/h = 16.6 $mmol_{CO2}\cdot g_{cat}^{-1}h^{-1}$

Table S2. Impact of Zr content on the structure and performance of the catalysts.

Catalyst	Surface composition (atom%)[a]				S_{BET} (m^2 g^{-1})	Catalytic activity[b]
	Co	Zr	B	O		
Co-B-O	7.7	-	3.6	88.7	18.8	0.9
Co-$Zr_{0.05}$-B-O	30.4	1.6	14.7	53.3	32.0	8.2
Co-$Zr_{0.1}$-B-O	34.7	5.1	18.9	41.3	92.4	10.7
Co-$Zr_{0.3}$-B-O	16.5	6.6	13.2	63.7	20.0	5.8

[a] The data were from XPS analysis, indicating the atomic percentage in total surface atoms.

[b] The unit of the activity is $mmol_{CO2}$ g_{cat}^{-1} h^{-1}, which was obtained at the same condition to that in Figure 1.

Table S3. The oxygen states of different catalysts determined by XPS characterization.

Catalyst	O 1s %		
	Lattice O	Surface O	B-O
Co-B-O	6.2	51.3	42.5
Co-$Zr_{0.05}$-B-O	45.5	41.7	12.8
Co-$Zr_{0.1}$-B-O	36.2	47.7	16.1
Co-$Zr_{0.3}$-B-O	35.0	40.5	24.5
Co-$Zr_{0.1}$-B-O-5r	33.9	51.6	14.5

The data of oxygen states were obtained from the XPS characterization of different catalysts.

Table S4. Result of the CO hydrogenation over the Co-$Zr_{0.1}$-B-O catalyst.

Product selectivity (%)				CO conversion (%)	Activity ($mmol_{CO}\ g_{cat}^{-1}\ h^{-1}$)
methane	ethane	propane	CO_2		
26.2	4.3	4.4	65.1	0.7	0.4

Conditions: catalyst 40 mg, cyclohexane 2 mL, 4 MPa CO, 4 MPa H_2, 180 ^oC, 12 h.

Reference

1. J. Masa, P. Weide, D. Peeters, I. Sinev, W. Xia, Z. Sun, C. Somsen, M. Muhler and W. Schuhmann, *Adv. Energy Mater,* 2016, **6**, 1502313.

2. U. B. Demirci and P. Miele, *Phys Chem Chem Phys,* 2010, **12**, 14651.

3. Y. Guo, S. Mei, K. Yuan, D. J. Wang, H. C. Liu, C. H. Yan and Y. W. Zhang, Low-Temperature CO_2 Methanation over CeO_2-Supported Ru Single Atoms, Nanoclusters, and Nanoparticles Competitively Tuned by Strong Metal–Support Interactions and H-Spillover Effect, *ACS Catal*. 2018, **8**, 7, 6203.

4. A. Karelovic and P. Ruiz, Mechanistic study of low temperature CO_2 methanation over Rh/TiO_2 catalysts, *J. Catal*., 2013, **301**, 141.

5. X. P. Guo, Z. J. Peng, A. Traitangwong, G. Wang, H. Y. Xu, V. Meeyoo, C. S. Li and S. J. Zhang, Ru nanoparticles stabilized by ionic liquids supported onto silica: highly active catalysts for low-temperature CO_2 methanation, *Green Chem.,* 2018, **20**, 4932.

6. C. I. Melo, D. Rente, M. N. da Ponte, E. Bogel-Lukasik and L. C. Branco, Carbon Dioxide to Methane Using Ruthenium Nanoparticles: Effect of the Ionic Liquid Media, *ACS Sustainable Chem. Eng.,* 2019, **7**, 11963.

7. T. Abe, M. Tanizawa, K. Watanabe and A. Taguchi, CO_2 methanation property of Ru nanoparticle-loaded TiO_2 prepared by a polygonal barrel-sputtering method, *Energy Environ. Sci.,* 2009, **2**, 315.

8. C. I. Melo, A. Szczepanska, E. Bogel-Lukasik, M. Nunes da Ponte and L. C. Branco, Hydrogenation of Carbon Dioxide to Methane by Ruthenium Nanoparticles in Ionic Liquid, *ChemSusChem,* 2016, **9**, 1081.

9. A. Karelovic and P. Ruiz, Improving the Hydrogenation Function of Pd/gamma-Al_2O_3 Catalyst by Rh/gamma-Al_2O_3 Addition in CO_2 Methanation at Low Temperature, *ACS Catal.,* 2013, **3**, 2799.

10. X. Shang, D. Deng, X. Wang, W. Xuan, X. Zou, W. Ding and X. Lu, Enhanced low-temperature activity for CO_2 methanation over Ru doped the Ni/$Ce_xZr_{(1-x)}O_2$ catalysts prepared by one-pot hydrolysis method, *Int. J. Hydrogen Energy*, 2018, **43**, 7179.

11. S. Tada, O. J. Ochieng, R. Kikuchi, T. Haneda and H. Kameyama, Promotion of CO_2 methanation activity and CH_4 selectivity at low temperatures over Ru/CeO_2/Al_2O_3 catalysts, *Int. J. Hydrogen Energy*, 2014, **39**, 10090.

12. C. Swalus, M. Jacquemin, C. Poleunis, P. Bertrand and P. Ruiz, CO_2 methanation on Rh/r-Al_2O_3 catalyst at low temperature: “In situ” supply of hydrogen by Ni/activated carbon catalyst, *Appl. Catal., B,* 2012, **125**, 41.
